# Book Reviews

**Published:** 1891-07

**Authors:** 


### Book Reviews.

Principles of Surgery. By N. Senn, M. D., Ph. D., Professor Principles of Surgery and Surgical Pathology, Rush Medical College, Chicago ; Surgeon to the Milwaukee Hospital, etc. Illu trated. F. A. Davis, Philadelphia, 1890.

The successful study and practice of any branch of the healing art require a thorough knowledge of the principles upon which it is based. The student who has mastered the principles of surgery will have no difficulty in applying his knowledge in practice (preface). It is essentially the *principles* of surgery that are discussed in this volume, such as regeneration of tissue, inflammation, pathogenic bacteria, suppuration, pyæmia, erysipelas, surgical tuberculosis, etc.; and these are handled in the masterly manner which we would expect from the distinguished author. No English work, so far as we know, so thoroughly covers the same ground. Its appearance is very opportune, and we are pleased to see that the verdict of book-reviewers has been uniformly favorable.

The Daughter ; her Health, Education, and Wedlock. By William M. Capp, M. D., Philadelphia, F. A. Davis.

This is a book of homely suggestions for mothers and daughters. To the latter it could be particularly valuable, inasmuch as it furnishes a plain and simple instruction on subjects of which girls are too often ignorant. There is not a physician who has not seen cases of inexcusable ignorance of this sort. The fault, after all, rests upon the mothers, and in educating these up to their duty such works as the above may accomplish a very wholesome result. If books such as this were more often read, and more closely followed, there would be more happiness in the family, but less work for the physician. For a practical, common sense talk on subjects too often ignored we commend this little work very heartily.

Literary Intelligence.—The *British Journal of Dermatology*, published by Mr. H. K. Lewis, Gower street, London, will in future be under the direction of a committee, consisting of Dr. H. G. Brooke, Dr. H. Radcliffe Crocker, Dr. T. Colcott Fox, Mr. Malcolm Morris, Dr. T. F. Payne and Dr. J. J. Pringle, the latter of whom will be the acting editor. As these gentlemen will have the coöperation of Mr. Jonathan Hutchinson, Dr. Robert Liveing, Dr. McCall Anderson, Dr. Allan Jamieson and Dr. Walter G. Smith, it will be thoroughly representative of British Dermatology.

